# KI-gestützte klinische Entscheidungsunterstützungssysteme: Herausforderungen und Potenziale

**DOI:** 10.1007/s00103-025-04092-8

**Published:** 2025-06-25

**Authors:** Maximilian Tschochohei, Lisa Christine Adams, Keno Kyrill Bressem, Jacqueline Lammert

**Affiliations:** 1 Institut für Künstliche Intelligenz und Informatik in der Medizin, Technische Universität München, School of Medicine and Health, TUM Klinikum, München, Deutschland; 2 Google Deutschland GmbH, München, Deutschland; 3 Institut für Diagnostische und Interventionelle Radiologie, Technische Universität München, School of Medicine and Health, Klinikum rechts der Isar, TUM Klinikum, München, Deutschland; 4 Institut für Kardiovaskuläre Radiologie und Nuklearmedizin, Technische Universität München, School of Medicine and Health, Deutsches Herzzentrum München, TUM Klinikum, München, Deutschland; 5 Frauenklinik und Poliklinik, Technische Universität München, School of Medicine and Health, Klinikum rechts der Isar, TUM Klinikum, TUM Klinikum, Ismaninger Str. 22, 81675 München, Deutschland

**Keywords:** Künstliche Intelligenz, Generative KI, Large Language Models, Shared Decision Making, Datenschutz, Artificial intelligence, Generative AI, Large Language Models, Shared decision-making, Data protection

## Abstract

Klinische Entscheidungsfindung ist komplex, zeitkritisch und fehleranfällig. Klinische Entscheidungsunterstützungssysteme (CDSS) sind computergestützte Anwendungen, die Ärzt:innen und andere Gesundheitsfachkräfte bei medizinischen Entscheidungen unterstützen – etwa in der Diagnosestellung, Therapieplanung oder Risikoeinschätzung. Unterschieden werden regelbasierte, wissensbasierte und KI-gestützte Ansätze von CDSS.

KI-gestützte CDSS gewinnen zunehmend an Bedeutung. Sie ermöglichen die Analyse großer Datenmengen und liefern evidenzbasierte Empfehlungen zur Unterstützung klinischer Entscheidungen. Zu den Herausforderungen zählen die Datenqualität, die Integration in klinische Abläufe und die Akzeptanz durch das Fachpersonal. Auch ethische und rechtliche Fragen, insbesondere bzgl. Datenschutz, bleiben kritisch. Derzeit finden KI-gestützte CDSS vor allem in der Radiologie (z. B. Lungenrundherde, Mammographie) und Kardiologie erfolgreich Anwendung. Sie tragen dort zur Verbesserung der Diagnosegenauigkeit und der Versorgungsqualität bei. Zukünftig können Chat- und Voicebots auf Basis großer Sprachmodelle (LLMs) im Bereich Shared Decision Making (SDM) eine wichtige Rolle spielen, indem sie Patient:innen gezielt informieren und in Entscheidungsprozesse einbeziehen.

Für eine effektivere, effizientere und patientenzentrierte Gesundheitsversorgung bieten KI-gestützte CDSS ein großes Potenzial. Voraussetzung dafür ist ein verantwortungsvoller Einsatz von KI, der ethische und rechtliche Anforderungen berücksichtigt. Eine passende Applikationsarchitektur kann diesen Anspruch unterstützen – etwa durch die Verknüpfung von KI-Modellen mit domänenspezifischen Daten, die Anonymisierung von Anfragen sowie die Validierung der generierten KI-Antworten.

## Abstract (en)

Clinical decision-making is inherently complex, time-sensitive, and prone to error. AI-enabled clinical decision support systems (CDSS) offer promising solutions by leveraging large datasets to provide evidence-based recommendations. These systems range from rule-based and knowledge-based to increasingly AI-driven approaches. However, key challenges persist, particularly concerning data quality, seamless integration into clinical workflows, and clinician trust and acceptance. Ethical and legal considerations, especially data privacy, are also paramount.

AI-CDSS have demonstrated success in fields like radiology (e.g., pulmonary nodule detection, mammography interpretation) and cardiology, where they enhance diagnostic accuracy and improve patient outcomes. Looking ahead, chat and voice interfaces powered by large language models (LLMs) could support shared decision-making (SDM) by fostering better patient engagement and understanding.

To fully realize the potential of AI-CDSS in advancing efficient, patient-centered care, it is essential to ensure their responsible development. This includes grounding AI models in domain-specific data, anonymizing user inputs, and implementing rigorous validation of AI-generated outputs before presentation. Thoughtful design and ethical oversight will be critical to integrating AI safely and effectively into clinical practice.

## Einleitung

Die Entscheidungsfindung im klinischen Alltag ist oft von hoher Komplexität geprägt. Behandelnde Ärzt:innen müssen dabei eine Vielzahl relevanter Faktoren berücksichtigen: Patientenhistorie, aktuelle Symptome, Laborwerte, bildgebende Verfahren, wissenschaftliche Erkenntnisse und Leitlinien, aber auch individuelle Bedürfnisse und Präferenzen der Patient:innen. Erschwerend kommt hinzu, dass medizinische Entscheidungen oft unter Zeitdruck und mit Unsicherheiten getroffen werden müssen. Mitunter sind nicht alle relevanten Informationen verfügbar oder der Gesundheitszustand der Betroffenen ändert sich rasch.

Diese Komplexität erhöht das Risiko für Fehlentscheidungen [1]. Zudem führt die stetig wachsende Menge an medizinischen Daten und Publikationen zu einer Informationsüberflutung, die es erschwert, den Überblick zu behalten und alle relevanten Informationen in Entscheidungen miteinzubeziehen. Insbesondere in Bereichen ohne etablierte medizinische Leitlinien müssen Kliniker:innen häufig eine Vielzahl an Dokumenten durcharbeiten, um fundierte Entscheidungen treffen zu können [2].

Klinische Fehlentscheidungen können schwerwiegende Folgen haben. Sie gefährden nicht nur die Patientensicherheit, sondern können auch zu vermeidbaren Komplikationen, längeren Krankenhausaufenthalten und erhöhter Mortalität führen [3]. Darüber hinaus tragen Fehlentscheidungen zu Ineffizienz und steigenden Kosten im Gesundheitswesen bei [4].

Um die Qualität klinischer Entscheidungen zu verbessern und die Patientensicherheit zu erhöhen, besteht ein zunehmender Bedarf an Unterstützungssystemen, die Ärzt:innen im Entscheidungsprozess begleiten. Künstliche Intelligenz (KI) bietet hier vielversprechende Ansätze. KI-gestützte klinische Entscheidungsunterstützungssysteme (Clinical Decision Support Systems, CDSS) sind in der Lage, große Datenmengen zu analysieren, Muster zu erkennen und evidenzbasierte Empfehlungen zu generieren, insbesondere wenn sie von einer strukturierten Implementierung von Leitlinien unterstützt werden. Sie können Ärzt:innen bei Diagnosefindung, Therapieplanung und Risikomanagement unterstützen und so zu einer effizienten und sicheren Patientenversorgung beitragen [5]. Ein weiteres Einsatzszenario KI-gestützter CDSS ist die Integration in Chat- und Voicebots, etwa im Zusammenhang mit Shared Decision Making (SDM). Hierbei werden medizinische Entscheidungen von Ärzt:innen und Patient:innen gemeinsam getroffen.

Dieser Artikel gibt einen Überblick über Herausforderungen und Potenziale KI-gestützter CDSS. Der Einsatz in verschiedenen medizinischen Fachbereichen wird beschrieben. Auf die Entscheidungsunterstützung für Patient:innen über Chat- und Voicebots wird eingegangen und abschließend eine Architektur für den wissensbasierten und datenschutzkonformen Einsatz von KI-Systemen zur Unterstützung von Patient:innen im Entscheidungsprozess vorgestellt.

## KI-gestützte klinische Entscheidungsunterstützungssysteme

CDSS sind Softwareanwendungen, die medizinisches Wissen mit Patient:innen-spezifischen Informationen kombinieren. Ziel ist es, Ärzt:innen bei der Entscheidungsfindung zu unterstützen, indem die Patientendaten analysiert, mit bestehendem Wissen abgeglichen und Empfehlungen oder Warnhinweise ausgegeben werden.

CDSS lassen sich in 3 verschiedene Typen unterteilen (Tab. 1): 1) *Wissensbasierte Systeme* nutzen umfangreiche Wissensdatenbanken und Inferenzmechanismen, damit Nutzende aus den Patientendaten Schlussfolgerungen ziehen können (z. B. UpToDate). 2) *Regelbasierte Systeme* verwenden zusätzlich aus Wissensdatenbanken abgeleitete, fest definierte Regeln und Algorithmen, die medizinisches Wissen abbilden und dadurch eine automatisierte Entscheidungsfindung ermöglichen. 3) *KI-gestützte Systeme* extrahieren Muster und Zusammenhänge aus großen Datensätzen und generieren Prognosen und Empfehlungen. Die Anwendungsgebiete von KI-gestützten CDSS sind vielfältig. In der Diagnostik können sie bei der Interpretation von Bildgebungsdaten oder der Identifizierung von Risikofaktoren unterstützen; in der Therapieplanung helfen sie bei der Auswahl der geeigneten Behandlungsstrategie und der Vorhersage des Therapieerfolgs [6].

**Tab. 1:** Arten von CDSS mit Vorteilen, Nachteilen und Beispielen

Art	Beschreibung	Vorteile	Nachteile	Beispiele
Regelbasierte Systeme	Basieren auf explizit definierten Regeln und Wenn-dann-Beziehungen, die von Expert:innen festgelegt werden	Einfach zu implementieren und zu verstehen; jede Entscheidung ist absolut nachvollziehbar	Können schnell sehr komplex werden; erfordern regelmäßige Aktualisierung; benötigen spezifische Eingabeformate	Antibiotikadosierungsempfehlungen basierend auf Patient:innencharakteristika und Infektionsart
Wissensbasierte Systeme	Verwenden eine durchsuchbare Wissensbasis (z. B. Symptome und medizinische Diagnosen), um Entscheidungen zu unterstützen	Können komplexes medizinisches Wissen abbilden; ermöglichen detaillierte Analysen und Erklärungen	Aufbau und Pflege der Wissensbasis ist aufwendig; Suche erfordert exakte Suchbegriffe; Empfehlungen für Laien oft schwer verständlich	Diagnoseunterstützungssysteme, die Symptome und Befunde mit Krankheitsbildern abgleichen (z. B. OncoKB, WebMD)
KI-gestützte Systeme	Nutzen Algorithmen, die aus Daten lernen, um Muster zu erkennen und Vorhersagen zu treffen	Können komplexe Zusammenhänge in großen Datensätzen analysieren; können sich an neue Daten anpassen; verarbeiten unstrukturierte Daten und Anweisungen	Erfordern große, qualitativ hochwertige Trainingsdaten; Empfehlungen können nicht ausreichend erklärt werden; unklare Haftung bei Fehlaussagen	Bildanalyse in der Radiologie zur Erkennung von Anomalien; Chatbot für Patient:inneninformationen

Grundlage für KI-gestützte CDSS sind KI-Modelle, die in der Regel über Verfahren des maschinellen Lernens (Machine Learning) trainiert werden. Diese Vorgehensweise ermöglicht Computern, aus Daten selbstständig zu lernen und Muster zu erkennen, ohne explizit programmiert zu werden. Dazu werden große Datenmengen, wie z. B. radiologische Bilddaten, von menschlichen Expert:innen annotiert [7]. Anschließend versucht ein Algorithmus, Muster in diesen Daten zu erkennen, die auf die Annotation schließen lassen. Anhand dieser Muster kann das KI-Modell in zuvor ungesehenen Daten selbstständig eine Annotation mit einer gewissen statistischen Wahrscheinlichkeit vorhersagen. Dieser Prozess wird auch „überwachtes Lernen“ genannt. „Unüberwachtes“ und „selbstüberwachtes Lernen“ sind neuartige Trainingsansätze, bei denen sich das KI-Modell selbst Strukturen aus Datensätzen erschließt. Deep Learning ist eine Unterform des Machine Learning, das auf neuronalen Netzen basiert und besonders gut geeignet ist, komplexe Zusammenhänge in großen Datensätzen zu analysieren. Natural Language Processing befasst sich mit der Verarbeitung und Analyse von natürlicher Sprache und ermöglicht es Computern, medizinische Texte wie Arztbriefe oder Forschungspublikationen zu verstehen. Die heute gängigen LLM, die in Lösungen wie „ChatGPT“ (OpenAI, San Francisco, Kalifornien, USA) oder „Google Gemini“ (Google LLC, Mountain View, Kalifornien, USA) verwendet werden, basieren auf einer Kombination aus Unsupervised Learning, Deep Learning und Ansätzen aus dem Natural Language Processing, um große Mengen von unstrukturierten Daten (Texte, Audio, Bilder und Videos) zu erschließen. Insbesondere LLM-gestützte KI-Systeme sollen durch dieses breite Grundlagenwissen, das viele medizinische Quellen einschließt, große Chancen bieten, um die medizinische Versorgung durch eine effektivere Unterstützung von Ärzt:innen zu verbessern [8].

## Herausforderungen KI-gestützter CDSS

Trotz ihres großen Potenzials stehen KI-gestützte CDSS bei der Implementierung in die klinische Routine vor verschiedenen Herausforderungen.

### Datenqualität und -verfügbarkeit.

Eine zentrale Herausforderung sind die Datenqualität und -verfügbarkeit. KI-Modelle benötigen große Mengen an hochwertigen Daten, um zuverlässige Ergebnisse zu liefern. Klinische Daten sind jedoch oft unvollständig, fehlerhaft oder nicht standardisiert, was die Trainings- und Evaluierungsprozesse von KI-Modellen erschwert [9]. Die Entwicklung von robusten Datenmanagementstrategien und die Etablierung von Standards für die Datenerfassung und -verarbeitung sind daher essenziell. Beim Einsatz von öffentlich verfügbaren KI-Modellen ist eine Konfiguration mit domänenspezifischem Expertenwissen erforderlich [10].

### Integration in klinische Arbeitsabläufe.

Eine weitere Herausforderung ist die Integration von CDSS in die klinischen Arbeitsabläufe. Die Systeme müssen benutzerfreundlich und intuitiv bedienbar sein, um von Ärzt:innen akzeptiert und effektiv genutzt zu werden. Die Interoperabilität mit bestehenden Krankenhausinformationssystemen ist ebenfalls entscheidend, um einen reibungslosen Datenaustausch und eine effiziente Integration in die klinischen Prozesse zu gewährleisten. Begleitende klinische Studien müssen durchgeführt werden, um zu belegen, dass der Einsatz von CDSS zu einer Verbesserung der Patientenversorgung führt [11].

### Akzeptanz und Vertrauen.

Eine geringe Akzeptanz und fehlendes Vertrauen in KI-basierte CDSS stellen weitere Hürden dar. Ärzt:innen könnten dem Einsatz von KI in der Medizin skeptisch gegenüberstehen, da sie einen Verlust an Autonomie und Kontrolle befürchten. Um die Akzeptanz zu fördern, ist es wichtig, die Funktionsweise und die Grenzen von KI-Systemen transparent zu kommunizieren und Ärzt:innen in den Entwicklungs- und Implementierungsprozess einzubeziehen. Auch die Patient:innen müssen über den Einsatz von KI informiert und ihre Bedenken müssen ernst genommen werden. Die Transparenz der Algorithmen und die Nachvollziehbarkeit der Empfehlungen sind ebenfalls wichtig, um Vertrauen in die Systeme zu schaffen. Schließlich sind die Validierung und Evaluation von KI-basierten CDSS unerlässlich, um ihre Wirksamkeit und Sicherheit zu gewährleisten [11, 12].

Neben technischen und anwenderbezogenen Herausforderungen sollten auch ethische und rechtliche Aspekte berücksichtigt werden. Insbesondere Datenschutz und Datensicherheit spielen eine zentrale Rolle. Die Frage, wer die Verantwortung für die Entscheidungen trägt, die auf Basis von KI-Systemen getroffen werden, sollte vor jedem Einsatz der Modelle in der Klinik geklärt werden [13]. Durch das Zusammenspiel aus der Verordnung über künstliche Intelligenz (KI-Verordnung) und der europäischen Medizinprodukte-Verordnung muss sowohl die Herstellung als auch der Einsatz von KI-CDSS sorgfältig geprüft und geplant werden [14]. Hier ist insbesondere die automatisierte Entscheidungsfindung ohne menschliche Intervention oder Prüfung als kritisch einzustufen.

## Status quo für den Einsatz KI-gestützter CDSS

Der Einsatz KI-gestützter CDSS nimmt in der Medizin kontinuierlich zu. Solche Systeme analysieren große Datenmengen, erkennen Muster und generieren evidenzbasierte Empfehlungen zur Unterstützung klinischer Entscheidungen. Eine zentrale Voraussetzung für ihre Integration ist die Interoperabilität mit bestehenden IT-Systemen. Standards wie DICOM (für Bilddaten) und HL7 FHIR (für Patientendaten) ermöglichen dabei einen reibungslosen Informationsaustausch mit Krankenhausinformationssystemen (KIS). Neben der Unterstützung von Fachkräften eröffnet KI auch neue Möglichkeiten im Bereich Shared Decision Making (SDM), etwa durch dialogbasierte Anwendungen. Abschließend werden Herausforderungen sowie eine Architektur für den verantwortungsvollen, datenschutzkonformen Einsatz von KI im Entscheidungsprozess skizziert.

## KI-gestützte CDSS werden von Ärzt:innen bereits erfolgreich eingesetzt

**Abb. 1:**
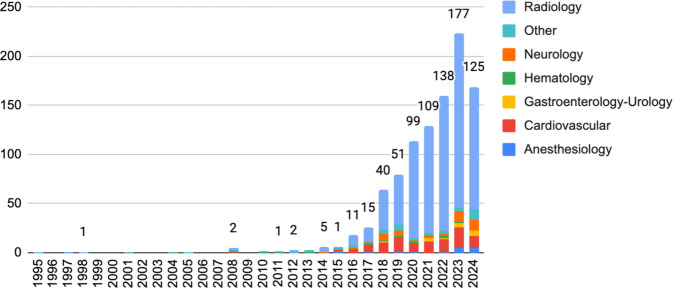
Anzahl der Zulassungen von KI-Algorithmen für den Klinikeinsatz durch die US-amerikanische Food and Drug Administration (FDA; [17])

Der Einsatz von KI-gestützte CDSS gewinnt in der medizinischen Praxis zunehmend an Bedeutung. In verschiedenen medizinischen Fachgebieten wurden bereits erfolgreich KI-gestützte CDSS implementiert, die nachweislich zu einer Verbesserung der Patientenversorgung beitragen [15, 16]. Darüber hinaus zeigt sich, dass die Anbindung solcher Systeme an telemedizinische Plattformen gerade in ländlichen Gebieten den Zugang zu Spezialdiagnostik verbessern kann. Damit die von KI generierten Empfehlungen nachvollziehbar werden, integrieren einige Projekte bereits erste Verfahren mit der „erklärbaren KI“ (Explainable AI, XAI). Die US-amerikanische Food and Drug Administration (FDA) hat bereits über 1000 KI-Algorithmen zum Einsatz freigegeben, davon 777 in der Radiologie [17]. Ein Blick auf die Zulassungszahlen zeigt einen starken Anstieg der Zulassungen seit 2015 (Abb. 1).

Vor allem in der Radiologie werden KI-Algorithmen zunehmend für die Analyse und Interpretation von radiologischen Bildern eingesetzt. Diese Algorithmen werden darauf trainiert, Muster in riesigen Datensätzen von Röntgenbildern, Computertomographien (CT) und Magnetresonanztomographien (MRT) zu erkennen und können so Anomalien wie Tumore, Frakturen, Gefäßveränderungen oder Entzündungen zu identifizieren. Ein konkretes Beispiel ist der Einsatz von KI zur Erkennung von Lungenrundherden in CT-Scans. Lungenkrebs ist eine der häufigsten Krebsarten weltweit und die Früherkennung ist entscheidend für die erfolgreiche Behandlung. KI-Systeme können CT-Bilder des Brustkorbs analysieren und selbst kleinste Lungenrundherde identifizieren, die für das menschliche Auge leicht zu übersehen sind. Durch die Markierung verdächtiger Bereiche auf den Bildern können Radiolog:innen diese gezielter untersuchen und so die Wahrscheinlichkeit einer frühzeitigen Diagnose erhöhen [18].

Auch in der Analyse von Mammographien werden CDSS zunehmend eingesetzt [19]. Die Systeme werden mit großen Datensätzen von Mammographieaufnahmen trainiert, um Anomalien und potenzielle Tumore zu detektieren. Eine hohe Datenqualität ist hier von entscheidender Bedeutung: Der DICOM-Standard stellt sicher, dass die Bilddaten einheitlich strukturiert vorliegen, was die KI-Analyse und den Vergleich zwischen verschiedenen Kliniken erleichtert. Dennoch bleibt zu beachten, dass Bias entstehen kann, wenn bestimmte Patientengruppen (z. B. unterschiedliche Brustgewebedichte, verschiedene Ethnien) in den Trainingsdaten nicht ausreichend repräsentiert sind. Die PRAIM-Studie („*PR*ospective, multicenter observational study of an integrated *AI* system with live *M*onitoring to support breast cancer screening“) mit über 460.000 Screening-Mammographien zeigte, dass KI-unterstützte Befundung die positive Prädiktion von Brustkrebserkennung um 17,6 % steigern kann bei gleichbleibender Recall-Rate [20]. Das System markiert verdächtige Areale und unterstützt so die radiologische Beurteilung. Besonders effektiv ist der KI-Einsatz bei der Zweitmeinung, wo der Arbeitsaufwand ohne Qualitätseinbußen reduziert werden kann. Die Technologie verbessert die Früherkennung von Brustkrebs und steigert die Effizienz der Screening-Programme. Die KI fungiert dabei als Unterstützungssystem für die radiologische Expertise.

Weiterhin hat KI in der Kardiologie Einzug gehalten und unterstützt Ärzt:innen bei der Diagnose und Behandlung von Herzerkrankungen. KI-gestützte Elektrokardiogramme (KI-EKGs) zeigen vielversprechende Ergebnisse bei der Erkennung von Erkrankungen wie linksventrikulärer Dysfunktion, asymptomatischem Vorhofflimmern und hypertropher Kardiomyopathie, manchmal sogar bevor erste Symptome auftreten [21]. Allerdings sind hier noch weitere Studien und Validierung erforderlich, um die Genauigkeit und Effektivität von KI-EKGs sicherzustellen – insbesondere im Hinblick auf ihren Einsatz in verschiedenen Bevölkerungsgruppen, die in den Trainingsdaten unterrepräsentiert sein könnten [22]. Um solchen Bias gezielt zu reduzieren, werden vermehrt prospektive, multizentrische Studien in diversen Patientenkollektiven initiiert. Diese sollen die Fairness und Repräsentativität der Algorithmen überprüfen, sodass KI-EKGs langfristig in unterschiedlichen Versorgungssettings sicher zum Einsatz kommen können.

Die erzielten Erfolge durch den Einsatz von KI-gestützten CDSS sind vielfältig. Studien zeigen, dass diese Systeme die Diagnosegenauigkeit verbessern, die Anzahl der Fehler reduzieren und Patient:innen-Outcomes optimieren können [15, 16]. Allerdings erzielen sie die besten Ergebnisse, wenn sie im Zusammenspiel mit geschulten Kliniker:innen eingesetzt werden [23].

## Chat- und Voicebots zur Entscheidungsunterstützung für Patient:innen

Shared Decision Making (SDM) ist ein patientenzentrierter Ansatz, bei dem Ärzt:innen und Patient:innen gemeinsam Entscheidungen über diagnostische und therapeutische Maßnahmen treffen. Dabei werden die individuellen Bedürfnisse, Präferenzen und Werte von Patient:innen berücksichtigt und in die Entscheidungsfindung einbezogen, was nachweislich zu besseren Patient:innen-Outcomes führt [24]. Die Implementierung von SDM erfordert eine intensive Schulung des medizinischen Personals und die Bereitschaft von Patient:innen zur Teilnahme, um sie über alle verfügbaren Behandlungsoptionen umfassend aufzuklären und sie bei der Abwägung von Nutzen und Risiken, aber auch bei der Wahrnehmung von Unsicherheiten zu unterstützen. Die deutsche Gesetzgebung (§ 630e BGB) unterstreicht die Bedeutung von Patientenautonomie und fördert den Einsatz von Entscheidungshilfen. Dennoch besteht in der klinischen Praxis eine erhebliche Diskrepanz zwischen den gesetzlichen Vorgaben und der tatsächlichen Umsetzung. Eine wesentliche Herausforderung bei der Implementierung von SDM ist sein Zeitaufwand. Komplexe Entscheidungsprozesse erfordern eine individuelle Zuwendung durch geschulte Fachkräfte sowie die Anerkennung von Patientenpräferenzen – auch dann, wenn diese nicht mit der ärztlichen Empfehlung übereinstimmen. Fehlt diese Unterstützung, greifen Patient:innen häufig auf nicht validierte Informationen aus dem Internet und den sozialen Medien zurück, die mitunter irreführend oder sogar gesundheitsschädlich sein können [25].

KI-gestützte CDSS bieten großes Potenzial, um SDM zu unterstützen und die Patient:innen aktiv in den Entscheidungsprozess einzubinden. Chat- und Voicebots können Patient:innen individuell informieren, ihnen helfen, ihre Präferenzen und Werte zu identifizieren und sie auf die Entscheidungsfindung gemeinsam mit ihren Ärzt:innen vorbereiten [26]. Gerade für Menschen, die weniger digital versiert sind oder keinen regelmäßigen Internetzugang haben, können Sprechstundenhilfen oder kurze Einweisungsvideos eine wertvolle Unterstützung bieten. Auf diese Weise wird die Teilhabe am SDM-Prozess auch für ältere oder sozial benachteiligte Patient:innen ermöglicht. Diese Systeme gehen auf die individuellen Fragen und Bedürfnisse der Patient:innen ein und vermitteln ihnen personalisierte Informationen, oft freundlicher und ausführlicher als die unter großem Zeitdruck leidenden Kliniker:innen [27]. Auch der Einsatz menschenähnlicher Avatare ist möglich, welche die Hemmungen von Patient:innen, mit KI-Systemen zu interagieren, weiter reduzieren können [28].

KI-gestützte CDSS für SDM erfordern den Einsatz großer Sprachmodelle (LLMs), wie sie auch in kommerziellen Chatbots wie ChatGPT oder Google Gemini zu finden sind. Einfache Textmodelle stoßen bei SDM an ihre Grenzen: Sie können zwar Absichten und Stimmungen aus Texten erkennen, allerdings liegen sie häufiger falsch und schöpfen ihre Antworten nur aus einer endlichen Menge vorgeschriebener Textbausteine. LLMs sind dagegen mit gewaltigen Datenmengen trainiert und dadurch in der Lage, den Kontext, die Absichten und die Gefühlslage aus einer Nachricht zu extrahieren und eine fast menschliche, empathische Antwort zu formulieren [29]. Dadurch können sie die Aussagen von Patient:innen präziser interpretieren und angemessen reagieren. Sie können außerdem komplexe medizinische Informationen in eine für Patient:innen verständliche Sprache übersetzen und dabei den individuellen Kenntnisstand, die kognitiven Fähigkeiten und die emotionalen Bedürfnisse der Patient:innen berücksichtigen [30, 31]. Einfache Textmodelle hingegen liefern oft nur generische Informationen, die für Patient:innen schwer verständlich oder nicht relevant sind. Um LLMs für SDM effektiv einsetzen zu können, müssen jedoch 2 große Herausforderungen überwunden werden: die Vermeidung von Falschaussagen und die datenschutzkonforme Verarbeitung von Patienteninformationen. Zukünftig könnten Ansätze wie „föderiertes Lernen“ (Federated Learning) zum Einsatz kommen, bei denen die KI-Modelle lokal in den Kliniken trainiert werden und nur die aggregierten Parameter an einen zentralen Server übermittelt werden. Weitere Verfahren wie Differential Privacy ermöglichen es, aus den geteilten Daten selbst keine Rückschlüsse auf einzelne Personen zu ziehen [32].

Wie alle KI-Modelle versuchen LLMs basierend auf einem Eingabewert (Nutzerfrage) einen Ausgabewert bzw. eine Sequenz von Ausgabewerten (KI-Antwort) vorherzusagen. Die Ausgabewerte lassen sich dabei über sogenanntes Hyperparameter-Tuning zwischen den statistisch wahrscheinlichsten Ausgabewerten und kreativen KI-Antworten konfigurieren. LLMs können deswegen eine plausibel klingende, aber inkorrekte Antwort erzeugen – eine sogenannte Halluzination. Bei der Verwendung eines kommerziellen Chatbots wie ChatGPT mit komplexen medizinischen Fragestellungen treten diese Falschantworten gehäuft auf [30, 31, 33]. Diese Falschaussagen lassen sich über die Verknüpfung des LLMs mit einer externen Wissensbasis deutlich reduzieren: Eine sogenannte Retrieval-Augmented-Generation-(RAG-)Architektur verbindet ein LLM mit einer kuratierten Wissensbasis – etwa klinischen Leitlinien oder wissenschaftlichen Artikeln – und stellt sicher, dass ausschließlich validierte Informationen ausgegeben werden: Gleichzeitig ermöglicht sie, die Herkunft der Antworten über Quellenangaben nachzuvollziehen [10, 34].

Die größere Herausforderung ist die datenschutzkonforme Verarbeitung von Patienteninformationen. Leistungsfähige KI-Modelle stellen sehr große Hardwareanforderungen an ihr Training und den anschließenden Betrieb [35]. Die leistungsfähigsten KI-Modelle sind proprietäre Angebote von Konzernen wie OpenAI, Google oder Anthropic und können nur über deren Cloud-Angebote bezogen werden. Manche KI-Modelle wie DeepSeek, Llama oder Mistral werden als sogenannte Open-Weight-Modelle veröffentlicht, das heißt, ihre trainierten Modellgewichte sind öffentlich verfügbar und können weiterverwendet oder angepasst werden, auch wenn Trainingsdaten und -code nicht offengelegt sind. Dadurch können sie auch von Dritten betrieben werden. Allerdings sind dafür Expertenwissen und unter Umständen teure Hardware erforderlich. Das in der Forschung beliebte Modell „DeepSeek-R1-Distill-Qwen-32B“ beispielsweise benötigt Hardware im Wert von mehreren 10.000 € für den Einsatz in Forschung und Produktion. Optimierungen wie Destillation oder Quantisierung können die Hardwareanforderungen reduzieren, erfordern aber tiefgreifendes Expertenwissen [36]. Das heißt, die meisten Forschenden und Kliniker:innen, insbesondere an kleineren Krankenhäusern, sind auf Cloud-Angebote angewiesen, die bei unsachgemäßer Benutzung Patienteninformationen außerhalb sicherer Kliniknetze verarbeiten. Während die KI-Verordnung in den nächsten Jahren Klarheit über den rechtskonformen Einsatz und die Nachvollziehbarkeit von KI-Modellen schafft, indem sowohl proprietäre KI-Modelle reguliert werden als auch klare Anforderungen an KI-Systeme gestellt werden, wird Datenschutz weiterhin eine große Herausforderung für den verantwortungsvollen Einsatz von KI-Systemen in der Medizin darstellen [37].

## Architektur für den datenschutzkonformen Einsatz von KI-Modellen in der Medizin

**Abb. 2:**
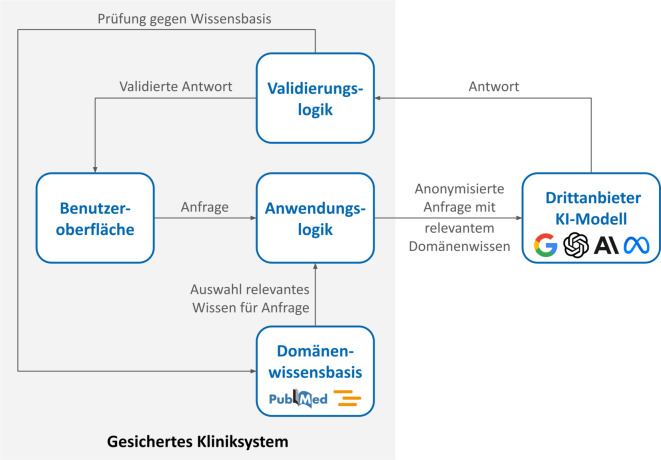
Architektur für ein datenschutzkonformes domänenspezifisches klinisches Entscheidungsunterstützungssystem (CDSS) zur Unterstützung der Entscheidungsfindung von Patient:innen im Rahmen des Shared Decision Making

Eine mögliche Architektur für einen wissensbasierten und datenschutzkonformen Einsatz von offenen und proprietären KI-Modellen in der Medizin basiert auf einer medizinischen Anwendungslogik, die der Arbeitsweise klinischen Personals nachempfunden ist (Abb. 2). Diese Anwendungslogik umfasst sowohl Anweisungen an das KI-Modell – sogenannte Prompts – als auch den Zugriff auf das klinische Fachwissen. Die Anweisungen definieren wichtige Anforderungen an das SDM-Verfahren: Das System ist auf eine informierende Funktion beschränkt und darf keine Beratung durchführen. Bei der Erkennung kritischer Situationen (beispielsweise Brustschmerzen mit Atemnot) werden Nutzer:innen aufgefordert, sich unmittelbar an medizinisches Fachpersonal zu wenden.

Die Wissensbasis wird ebenfalls im Kliniksystem aufgebaut und enthält Informationen, die für die Anwendung erforderlich sind, wie beispielsweise medizinische Leitlinien. Basierend auf der Nutzeranfrage wird über die Anwendungslogik nach einem RAG-Verfahren das relevante Wissen ausgewählt und mit dem Prompt kombiniert. Eine zentrale Funktion der Anwendungslogik liegt in der Pseudonymisierung und der möglichen Anonymisierung von Patientendaten, sowohl von direkt als auch von indirekt identifizierenden Informationen, um das Risiko einer Reidentifizierung zu minimieren. Direkte Identifikatoren (z. B. Name, Patientennummer, Geburtsdatum, Adresse) werden entfernt oder durch valide, aber nicht zurückverfolgbare Pseudonyme ersetzt. Indirekte Identifikatoren (z. B. exaktes Alter, seltene Erkrankungen, spezifische medizinische Details, detaillierte Zeitangaben zu Behandlungen) werden durch Umwandlung exakter Werte in breitere Kategorien anonymisiert. Die eingesetzten Techniken folgen dem Prinzip der Datensparsamkeit. Ziel ist es, die für die RAG-Anwendung notwendigen Informationen bereitzustellen (z. B. Altersgruppe, Krankheitsbild) und gleichzeitig den höchstmöglichen Schutz der Patientendaten gemäß der Datenschutzgrundverordnung (DS-GVO) und relevanten medizinischen Datenschutzstandards sicherzustellen. Das KI-Modell selbst bleibt austauschbar, da sich die heute angebotenen KI-Modelle nicht wesentlich in ihrer Funktionsweise und ihren Fähigkeiten im medizinischen Kontext unterscheiden, insbesondere wenn Techniken wie RAG eingesetzt werden [38].

Eine mit der RAG-Architektur kombinierte Validierungslogik sorgt dafür, dass die generierten Informationen korrekt und nachvollziehbar sind. Dazu wird die Antwort des KI-Modells mit der zugrunde liegenden Wissensbasis abgeglichen, um offensichtliche Falschinformationen vor der Übermittlung an die Nutzenden zu identifizieren. Zum Einsatz kommt dabei ein sogenannter LLM-as-a-Judge-Ansatz, bei dem speziell angepasste große Sprachmodelle unabhängig eine Überprüfung der Antwort gegen die ursprüngliche Datenbasis vornehmen. In Evaluierungen zeigen sich dabei Ergebnisse, die mit den Bewertungen menschlicher Expert:innen vergleichbar sind [39].

Die Qualitätssicherung wird durch eine stichprobenhafte Überprüfung der Antworten durch klinisches Fachpersonal (Human-in-the-Loop) gewährleistet. Darüber hinaus sollten regelmäßige Audits etabliert werden, um zu kontrollieren, ob Pseudonymisierungs- und Anonymisierungsprozesse korrekt angewendet werden. Externe Zertifizierungen (z. B. nach ISO 27001) können zusätzlich das Sicherheitsniveau bestätigen und die kontinuierliche Weiterentwicklung der Systeme fördern.

Abb. 2 stellt die beschriebene Architektur schemenhaft dar. Die Architektur bietet das Potenzial, Patient:innen bei der Entscheidungsfindung im Rahmen des SDM zu unterstützen, das klinische Personal zu entlasten und zugleich die Einhaltung aktueller und absehbar zukünftiger regulatorischer Vorgaben sicherzustellen.

## Fazit und Ausblick

KI-gestützte CDSS verfügen über ein erhebliches Potenzial zur Verbesserung der medizinischen Versorgung. Durch die Analyse großer Datenmengen, die Erkennung komplexer Muster und die Generierung evidenzbasierter Empfehlungen können sie Ärzt:innen in der Diagnose, Therapieplanung und im Risikomanagement wirkungsvoll unterstützen. Aktuelle Anwendungsfelder reichen von der Radiologie über die Kardiologie bis hin zur personalisierten Onkologie. Besonders vielversprechend ist der Einsatz von KI-gestützten CDSS im Bereich SDM. Chat- und Voicebots auf Basis großer Sprachmodelle können Patient:innen angepasst an ihr Wissens- und Sprachniveau informieren, ihre Präferenzen erfassen und sie auf die Entscheidungsfindung gemeinsam mit ihren Ärzt:innen vorbereiten.

Der Einsatz solcher Systeme bringt jedoch vielfältige Herausforderungen mit sich – insbesondere in Bezug auf technische Anforderungen wie Datenqualität, Interoperabilität und Systemintegration, aber auch hinsichtlich ethischer, rechtlicher und organisatorischer Fragestellungen. Um diesen Anforderungen gerecht zu werden, wurde in diesem Beitrag eine Architektur für den wissensbasierten und datenschutzkonformen Einsatz von KI in der Medizin vorgestellt. Diese basiert auf einer medizinischen Anwendungslogik, die zentrale Aufgaben übernimmt: Sie strukturiert die Anweisungen an das KI-Modell, steuert den Zugriff auf valide medizinische Wissensquellen und ermöglicht die Pseudonymisierung sensibler Patient:innendaten vor der Verarbeitung. In Kombination mit einer RAG-Architektur, die externe Wissensquellen dynamisch einbezieht, und einer Validierungslogik, die medizinisch-fachliche Korrektheit sowie Nachvollziehbarkeit der generierten Inhalte sicherstellt, entsteht ein robustes Gesamtsystem.

Mit dem Fortschritt von KI-Technologien und der zunehmenden Verfügbarkeit hochwertiger klinischer Daten werden solche Systeme in der Gesundheitsversorgung an Bedeutung gewinnen. Ihr Erfolg hängt jedoch maßgeblich davon ab, dass sie verantwortungsvoll gestaltet und in die Versorgungsrealität integriert werden – unter Berücksichtigung von Transparenz, Datenschutz und dem Vertrauen aller Beteiligten. Nur so kann das Potenzial KI-gestützter CDSS langfristig zum Wohl der Patient:innen ausgeschöpft werden.
